# Implementation of the epilepsy center of excellence to improve access to and quality of care – protocol for a mixed methods study

**DOI:** 10.1186/1748-5908-9-44

**Published:** 2014-04-09

**Authors:** Mary Jo Pugh, Luci K Leykum, Holly J Lanham, Erin P Finley, Polly H Noël, Katharine K McMillan, Jacqueline A Pugh

**Affiliations:** 1 South Texas Veterans Health Care System, University of Texas Health Science Center at San Antonio, 7400 Merton Minter BLVD, San Antonio, TX 78229, USA; 2 VA Epilepsy Centers of Excellence, 7400 Merton Minter BLVD, San Antonio, TX 78229, USA

**Keywords:** Epilepsy, Relational Coordination, Veterans Health Administration (VA)

## Abstract

**Background:**

To address the growing problem of epilepsy among aging Veterans and younger Veterans who have experienced a traumatic brain injury (TBI), the Veterans Health Administration (VA) has implemented 16 Epilepsy Centers of Excellence (ECOE) to assure increased access to high quality of care for Veterans with epilepsy. Each ECOE consists of a network of regional hubs to which spoke facilities refer Veterans for subspecialty treatment. The ECOEs are expected to improve access to and quality of epilepsy care through patient care, consultation and education. This study aims to: evaluate the effectiveness of the ECOE structure by describing changes in the quality of and access to care for epilepsy before and after the ECOE initiative using QUality Indicators in Epilepsy Treatment (QUIET Indicators); describe associations between changes in the structure and processes of care and Relational Coordination (RC), a model of task-oriented communication that has been shown to play a role in implementation science; and determine if variations in care are related to levels of RC.

**Methods:**

This four-year comparative case study uses a mixed-methods approach. We will use VA inpatient, outpatient, pharmacy, and chart abstraction data to identify changes in the quality of and access to epilepsy care in the VA between Fiscal Year 2008 and Fiscal Year 2014. Qualitative and survey methods will be used to identify changes in the structure and processes of epilepsy care and RC over the course of the study. We will then link data from the first two objectives to determine the extent to which quality of and access to epilepsy care is associated with RC using multivariable models.

**Discussion:**

This innovative study has the potential to improve understanding of hub-and-spoke model effectiveness, VA epilepsy care, and models of epilepsy specialty care more globally. Moreover, it contributes to implementation science by advancing understanding of the role of RC in the context of a major transformation in the structure of care delivery in a national integrated healthcare system.

## Background

Epilepsy is a growing problem in the Veterans Health Administration (VA) patient population among aging Veterans of Vietnam and younger Veterans of Operation Enduring Freedom/Operation Iraqi Freedom (OEF/OIF) who have experienced a traumatic brain injury (TBI), the biggest risk factor for new-onset epilepsy in younger adults. To address this concern, the VA has implemented Epilepsy Centers of Excellence (ECOE) to assure increased access to high quality of care for Veterans with epilepsy.

**Figure 1:**
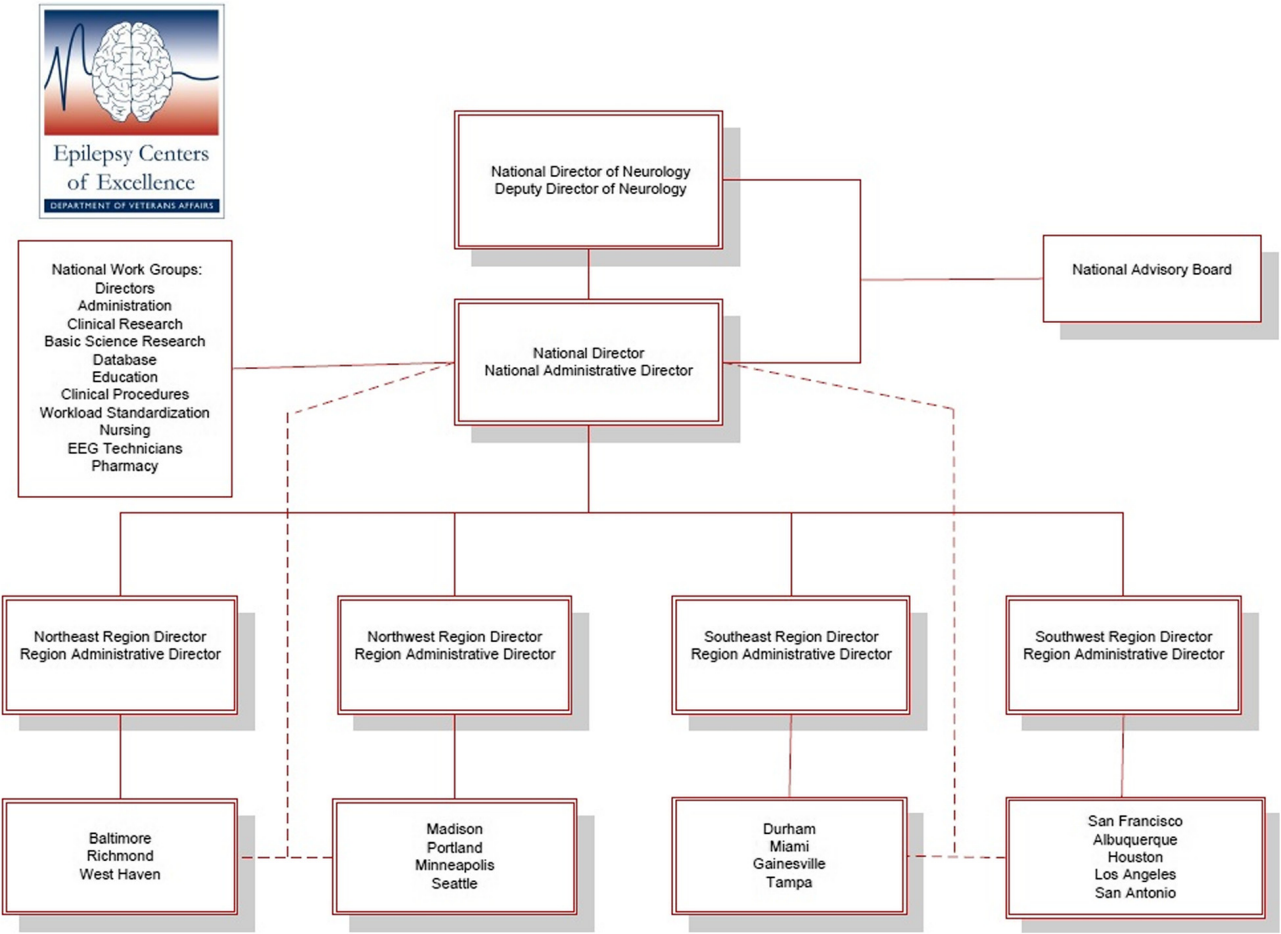
Epilepsy centers of excellence organizational structure.

Mandated by the Veterans Mental Health and Other Care Improvements Act of 2008 (PL110-387), the ECOE is comprised of 16 VA ‘hub’ facilities geographically dispersed in four regions to which ‘spoke’ facilities that lack specialty care and/or necessary diagnostic equipment may refer Veterans for specialty care within their region. Hubs within each region are linked to an accredited medical school and a Polytrauma Rehabilitation Center (PRC), which treats Veterans with TBI who at risk of developing post-traumatic epilepsy (PTE). This hub and spoke network is led by a national director with regional directors overseeing hubs within each region (Figure 1). This network structure was designed to improve access to epilepsy specialty care to Veterans across the country. Ideally, access is improved directly by providing a referral network for Veterans from remote sites and indirectly by providing outreach education and telehealth opportunities for patient consultation.

The ECOE’s program goals are to establish a national system of care that functions as a center of excellence in research, education, and clinical care activities in the diagnosis and treatment of epilepsy. ECOE directors are tasked with developing a national consortium of providers at spoke facilities lacking requisite resources to ensure better access to diagnosis, research, clinical care, and education. The ECOE also disseminates up-to-date information on epilepsy and epilepsy care for Veterans via written and web-based modalities. Thus, the goals of this network are to improve access to VA epilepsy specialty care and improve the quality of care provided to those who receive chronic disease management in primary care and general neurology settings.

### Quality of care: epilepsy

The limited research on the quality of epilepsy care in the United States suggests room for improvement both within and outside the VA. Our prior work, among the earliest example of epilepsy quality assessment in the elderly [1-3] found that over half of older Veterans with new-onset epilepsy received sub-optimal anti-epileptic drugs (AEDs), according to recommendations on appropriate medications for geriatric patients [1,2]. Studies in non-VA settings found that the time from epilepsy diagnosis to referral for surgery for temporal lobe epilepsy patients who fail two or more first-line AEDs remains relatively unchanged at 18 years [4], despite recommendations for referral after only one year of AED failure by the National Association of Epilepsy Centers [5]. Poorly controlled epilepsy is associated with increased cognitive dysfunction, mortality, injury and lower quality of life [6-17], and accounts for 80% of the cost of treating epilepsy [18]. Thus, assessing and improving the quality of epilepsy care has implications for patient outcomes and healthcare costs.

We developed the first quality measures for the care of adults with epilepsy, the QUality Indicators for Epilepsy Treatment (QUIET), for use in primary care and general neurology settings using an evidence-based expert consensus process (Appendix) [19]. These measures were used as a foundation for the American Academy of Neurology Epilepsy Quality Indicators [20]. Our work in non-VA settings found that the concordance between recommended and actual care provided ranged from 0% to 99%, with 44.2% of recommended care processes being performed. Data indicated similar proportions of concordance for initial seizure assessment (42%), initial epilepsy diagnosis and treatment (44%), chronic epilepsy care (46%), and aspects of care unique to women (36%). More recently, we adapted these indicators to the unique setting of the integrated VA healthcare system (QUIET-VA). We do not know whether Veterans living with epilepsy are receiving high quality care as defined by the QUIET-VA measure, or if access to care and quality of care have improved since implementation of the ECOE initiative.

### The role of relational coordination

In the Center of Excellence (COE) model of care delivery, access and quality are dependent upon the interconnections between individuals working to facilitate care in the hub and spoke system [21]. For example, information must flow effectively between hub and spoke for care to be timely, coordinated and appropriate for the patient. Thus, access and quality are properties that emerge from the hub and spoke system [22]. Assessing the impact of the ECOE initiative on access and quality requires an assessment of the interconnections and interdependencies among the hubs and spokes [23].

The ECOE model is intended to improve coordination among members of the epilepsy care team. For example, greater interaction of epilepsy specialty providers in team meetings and patient consultation may facilitate an awareness of how their work relates to the overall goals of the patient and to others in the epilepsy patient care team. Educational outreach may also increase interaction of epileptologists with primary care and general neurology providers, stimulating a sense of alignment and shared knowledge regarding their role in providing high quality epilepsy care.

Studies over the past decade indicate that the relationships, interactions, communication patterns, and flow of information among the individuals within the healthcare team are important to its functioning [21,24-26]. Simply changing structures and processes does not always lead to better outcomes [27,28]. Rather, changes in the organization and delivery of care that lead to increasing interconnections and to timely, accurate, problem-solving communication should generate better quality of care and patient outcomes. Relational Coordination (RC), mutually reinforcing interactions between communication and relationships within a team for the purpose of task integration [29], has been shown to be an important predictor of a team’s or organization’s ability to provide high quality care [30-33]. RC describes and measures cross-role or cross-task shared knowledge, shared goals and mutual respect among team members as well as the frequency, timeliness, accuracy, and problem-solving orientation of communication among team members. Furthermore, recent work suggests that team leaders play an essential role in promoting healthy communication, relationships, and learning among work groups that appear to be essential for coordinating and achieving complex goals [34,35].

In a setting such as the ECOEs where goals are defined broadly and leaders are allowed flexibility in achieving those goals, we believe Relational Coordination will have a significant influence on the quality of care provided.

### Hypotheses and objectives

We hypothesize that ECOE implementation would be associated with significant improvements in the quality of epilepsy care in such areas as: a) Veterans with newly diagnosed epilepsy, b) Veterans with chronic epilepsy, c) referrals, and d) epilepsy surgery as evidenced by a higher proportion of recommended processes of care delineated in the QUIET measures being performed for Veterans with epilepsy. Based on the RC framework, we propose that changes in the organization and delivery of care (*e.g*., broaden the membership of epilepsy care team, use of care pathways) which promote timely, accurate, and problem-solving communication among the epilepsy care team will lead to higher quality and more efficient epilepsy care.

Objective 1: Describe changes in quality of and access to epilepsy care before and after the ECOE initiative. We expect that the ECOE implementation will lead to significant improvements in the quality of epilepsy care as evidenced by a higher proportion of recommended processes of care delineated in the QUIET measures being performed.

Objective 2: Describe the associations between changes in the structure/ processes of care implemented by the four geographic ECOEs and RC among epilepsy care team members in each ECOE region. We expect that the ECOE regions that enact strategies that enhance RC (*e.g*., video case-conferences between hub and spoke physicians) will have higher RC scores than ECOEs that rely on traditional referral modalities (*e.g*., electronic consults) between facilities.

Objective 3: Determine whether variations in quality of and access to VA epilepsy care among ECOEs are associated with variations in RC. We expect that ECOE regions with higher RC scores will have higher QUIET-VA indicator performance ratings.

## Methods

### Study design overview

We are conducting a four-year comparative case study using a mixed-methods approach that incorporates strategies from patient safety/process improvement and implementation science [27,36]. We are using VA inpatient, outpatient, pharmacy, and chart abstraction data to identify changes in quality of and access to epilepsy care in the VA between FY08-FY14 (Objective 1). Qualitative and survey methods will be used to identify changes in the structure and processes of epilepsy care and RC over the course of the study (Objective 2). We will then link data from the first two objectives to determine the extent to which quality of and access to epilepsy care is associated with RC using multivariable models (Objective 3).

### Study population

Veterans meeting criteria for epilepsy using our previously validated epilepsy identification algorithm comprise the population for quality assessment (Objectives 1 and 3). Briefly, individuals with a diagnosis indicative of epilepsy (ICD-9-CM codes 345, 780.39, 649.4) who have concomitant use of an AED will be included. Using algorithms and techniques developed in previously funded VA studies, we will classify Veterans’ epilepsy status as being New-Onset, Chronic or New to VA [2]. We will also use administrative databases to classify epilepsy status as poorly controlled if they receive emergency or hospital care with a primary diagnosis of seizure/epilepsy, use two or more AEDs, switch AEDs, or have a designation of intractability in the epilepsy specific diagnosis.

Using the overall VA patient epilepsy population, we will first identify random samples of individuals from each geographic ECOE stratified by onset (new, chronic) and severity (controlled, poorly controlled) in FY08 (N = 1,600), FY12 (N = 1,600), and FY14 (N = 1,600) for comparison of pre- and post-implementation of ECOE. The QUIET-VA measure (Appendix) will be used to identify quality of VA epilepsy care through medical chart abstraction (Additional file 1) in the year prior to ECOE implementation (FY08) and the years post-implementation (FY12, FY14). Secondary analyses will use population-based administrative data to assess broad measures of access to neurology and epilepsy specialty care to determine if access to epilepsy specialty care improved over the study period.

For Objective 2, we will sample individuals involved in the care of Veterans with epilepsy in select VA facilities, sampling each hub and spoke, and conduct an in-depth study in each hub-spoke system.

### Data sources

For Objective 1, we will employ three datasets to obtain information about inpatient/outpatient medical encounters, medication, and detailed information about care processes (Tables 1 and 2).

**Table 1:** Outcome measures, data sources, and definitions for Objective 1

**Type of measure**	**Data source(s)**	**Data used to define**
**Quality**		
QUIET-VA	VistA Web	Medical chart progress notes, problem list, screening, tests, medications, etc.
**Access**		
Epilepsy specialty care	PTF	Inpatient bedsection 11 (Epilepsy center) Clinic Stop: 315456 Epilepsy clinic **FY11 onward:** 345 Epilepsy center of excellence; other epilepsy stop codes: 345188, 345185, 345714, 345720
	OPC	
Epilepsy surgery, procedures, monitoring	PTF (OPC for Day of Surgery Admission) inpatient encounter files	ICD9A
		CPT codes
General neurology care	PTF	Inpatient bedsection: 10 Neurology, 34 geriatric evaluation neurology
	OPC	Clinic stop: 315 neurology clinic

OPC: outpatient clinic PTF: patient treatment file; VistA Web: Veterans Health Information Systems Technology and Architecture Web.

**Table 2:** Covariates for Objective 1 Analyses

**Type of measure**	**Database**	**Data used to define**
Socio-demographic variables	OPC, PTF	Age, sex, race, marital status
Clinical characteristics		
Number of unique drugs	Pharmacy	VA product; total number of oral, injectable, transdermal drugs in previous year.
Physical comorbidities	OPC, PTF	Traumatic brain injury, migraine/ headache, brain tumor, dementia, cerebrovascular disease, cardiac conditions, hypertension, other neurological disorders, obesity
Mental health comorbidities	OPC, PTF	Substance use disorder, schizophrenia, depression, bipolar disorder, post-traumatic stress disorder, anxiety
Distance to Hub/ Spoke	OPC	ZIP variable

OPC: outpatient clinic; PTF: patient treatment file.

Medical SAS Datasets: National administrative data for VA-provided healthcare are stored as Medical SAS Datasets in the VA national data repository in Austin, TX. These data repositories are updated daily with inpatient and outpatient encounter data from VA clinical information systems and include patient demographic information, date and type of care (clinic code for outpatient or bed section for inpatient care), up to 10 diagnoses, procedures, and provider type; inpatient files also include length of stay. Secondary analyses will examine access to epilepsy specialty care and epilepsy surgery.

VA Pharmacy Benefits Management (PBM) Data: We will use the outpatient pharmacy database, which contains prescription information for individual VA patients receiving medications from the VA, to identify individuals receiving AEDs (for epilepsy algorithm) and identify specific medications that are of interest for QUIET VA measure (*e.g*., warfarin, folate).

Medical chart abstraction: To examine the quality of epilepsy care as defined by the QUIET VA measure, we will perform medical chart abstractions for individuals identified above (Additional file 1). Two chart abstractors will use this tool, independently reviewing 10 charts; disagreements will be resolved by consensus. After there is 90% agreement on abstracted items, chart abstractors will perform remaining abstractions using the standardized chart abstraction form. In addition to quality, we will identify individuals who have epilepsy surgery, extended video EEG monitoring, or other procedures outside the VA.

Data for Objective 2 will be derived from surveys of epilepsy care teams from each selected facility (Table 3) and semi-structured interviews conducted with staff at selected facilities (Table 4). Semi-structured interviews will ask participants to articulate changes in the care processes. Moreover, archival documents generated by the ECOE (*e.g*., annual reports, minutes) and observations of meetings conducted by various ECOE groups (*e.g*., Directors, Basic Research) will be analyzed to examine the emerging impact of changes in the ECOE system.

**Table 3:** **Relational Coordination dimensions**[29]

**Dimension**	**Survey Item**
Frequent communication	How **frequently** do you communicate with people in each of these groups about epilepsy patients?
Timely communication	Do people in these groups communicate with you in a **timely** way about epilepsy patients?
Accurate communication	Do people in these groups communicate with you **accurately** about epilepsy patients?
Problem-solving Communication	When a problem occurs with epilepsy patients, do the people in these groups work with you to **solve the problem**?
Shared goals	How much do people in these groups **share your goals** regarding epilepsy patients?
Shared knowledge	How much do people in each of these groups **know** about the work you do with epilepsy patients?
Mutual respect	How much do people in these groups **respect** the work you do with epilepsy patients?
Communication modalities	Which of the following communication vehicles do you use when sharing information about epilepsy patients with the primary care providers (Check all that apply): phone, fax, email, text messaging, instant messaging, face-to-face meetings, video conferencing/meetings, tele-conferencing/meetings (audio), formal meetings, informal meetings, electronic consult for epilepsy, notes in the electronic medical records.

**Table 4:** Areas of exploration for key informant interviews

**Area of exploration**	**Specific information sought**	**Sample questions**
Type of epilepsy care	Type of epilepsy care provided by the facility	What changes to the type of care you provide have you noticed since ECOE initiative?
Organization of care	Clinical work flow at the site, *e.g*., in the epilepsy clinic, neurology clinic (for Spoke facilities without epileptologist), or ECOE	What is the structure of the epilepsy care team? How has that changed since ECOE initiative? How have the roles of individuals on the epilepsy care team changed since ECOE initiative?
Referral	Formal policies	Describe any formal changes that have been implemented since the ECOE initiative, including clinical pathways.
	Clinical work flow and communication involved in referrals	How are Spoke-to-Hub Referrals handled? Who initiates communication from the spoke? Who receives communication at the Hub?
		How are Hub to Hub Referrals handled?
	Satisfaction with referral processes	How satisfied are you with the current referral processes and why?
Use of technology	How is information technology (IT) being used to manage clinical work flow?	Describe strategies that have been developed to enhance access to epilepsy specialty care.
		Describe strategies that have been developed to improve the quality of epilepsy care for Veterans.
		Do you have plans to incorporate new IT tools/ modify existing tools to manage clinical work flow/ referral?
Educational approach	Types of education being offered at the ECOE	Describe the educational mission with regard to teaching fellows.
		Describe approach for educational outreach.
General impact of ECOE initiative	Most salient changes from the perspective of clinicians/administrators	Describe the most important changes that have resulted from the ECOE care structure.
	Barriers/facilitators	Describe any barriers you have encountered.
	Other unexpected changes	Describe any other unexpected changes or consequences you have experienced.
Other changes	Other changes in the field unrelated to the ECOE initiative	Describe any changes in the field of epilepsy care that you believe may have an influence on the quality of epilepsy care or access to epilepsy care within or outside the VA.

### Measures

Epilepsy status: Based on our algorithm, individuals who meet inclusion criteria for epilepsy and who have no use of antiepileptic drugs the year prior to the initial diagnosis (or who receive a new AED after the initial seizure) will be classified as having New-Onset epilepsy. Individuals with prior evidence of epilepsy will be classified as having Chronic epilepsy. Veterans without prior data will be classified as New to VA. The epilepsy severity described above will be used to identify individuals in the database who are more likely to have poorly controlled epilepsy (*i.e*., individuals who receive emergency or hospital care with a primary diagnosis of epilepsy and/or individuals receiving two or more AEDs after new diagnosis).

Quality of epilepsy care: We will first identify the completion of recommended care based on the QUIET-VA measure (Additional file 1) for each individual. Quality of epilepsy care will be quantified as the proportion of recommended processes of care that were provided for each patient. We will examine overall quality, using the same categories used in our prior study: new seizure, initial diagnosis and treatment, chronic epilepsy care, and care unique to women.

Access to VA neurology and epilepsy care: Secondary analyses will also examine access to epilepsy specialty care and neurology care first using National VA administrative data. The primary measure, Epilepsy Specialty Care, will identify individuals who receive any inpatient or outpatient Epilepsy Specialty care based on clinic codes, ICD-9A and CPT codes.

Patient characteristics: In addition to our primary outcomes, we will identify patient characteristics for use as covariates/case-mix adjustments for multivariable models, and distance from the hub/spoke as a covariate (Table 2).

Relational Coordination: The RC survey includes questions that examine seven dimensions (Table 3) of how individuals interrelate (Objective 2) [29]. We will also include an examination of communication modalities (*e.g*., face to face communication, email; Computerized Patient Record System [CPRS]) within the RC survey. Items are rated by participants on a 5-point scale indicating the frequency to which each dimension exists in their care setting (*e.g*., frequency: 1 = Never, 5 = Constantly) [29].

RC scores are first calculated for each individual by summing the scores of all roles (*e.g*., neurologist, epileptologist, registered nurse, etc.) for each dimension (*e.g*., frequent communication) and then dividing by the number of responses. The overall RC score for each participant is derived by calculating the mean of the seven individual scores (range 1–5) [29].

RC scores at the facility level are calculated for each functional group (e.g., neurologist, epileptologist, nurse practitioner) by calculating the mean of each dimension for all members of the functional group, and then a facility RC mean and an overall ECOE RC (hub and spokes) mean. The primary analyses will use the regional ECOE RC mean score, and secondary analyses will examine variation in RC scores among functional groups and hubs vs. spokes [29].

Semi-structured interviews: Semi-structured interviews will be used to obtain richer details regarding the organization of care and specific innovative approaches used by epilepsy care teams in each ECOE to improve the quality of and access to care (Table 4). We will use data collected for the previous objectives to complete Objective 3 using a mixed methods approach for studying the ECOE initiative’s impact on access to and quality of epilepsy care. Each data type will first be analyzed independently to detect key patterns and associations among the variables in both the qualitative and quantitative data. Quantitative data will be analyzed using statistical methods to better understand the impact of the ECOE initiative on access and quality. Qualitative data will be analyzed using constant comparative analysis and open coding methods to generate new understandings of how the contextual nuances in the hubs and spoke facilities during and after the ECOE implementation are associated with access and quality.

Once we have generated the key findings from each data type, we will turn our attention to integrating the findings from these diverse data using a triangulation protocol [37]. We define triangulation as a process of studying a phenomenon using different methods to gain a more complete picture. The goal of this integration is to generate knowledge that goes beyond what is suggested from independent analysis of the qualitative and quantitative data [38]. To do this, we will list the findings from each data analysis effort together and consider where the findings from each method agree, where they provide complementary insights on the same topic, or where they contradict each other, developing meta-themes that go beyond simply reporting the findings from the administrative data, surveys and interviews. Final interpretations that result from this process will arise from the patterns in the data itself as well as the application of our primary theoretical framework, Relational Coordination.

### Study status

Recruitment for survey participation began on March 7, 2013. Survey data was collected from March 7, 2013, and concluded April 30, 2013. Round 1 interviews commenced March 5, 2013, and are ongoing through March 5, 2014. Round 2 data collection is slated to be conducted from September, 2014 through August, 2015.

## Discussion

Despite the increasing use of COE models within the VA, research examining this model’s effectiveness in terms of its impact on patient outcomes is sparse. This innovative study has the potential to improve understanding of hub-and-spoke model efficacy, VA epilepsy care, and models of epilepsy specialty care more globally. Moreover, it contributes to implementation science by advancing understanding of the role of RC in the context of a major transformation in the structure of care delivery in a national integrated healthcare system.

Even though our project is focused on epilepsy care, COE models are commonly used to provide care for individuals with disease states that are less common (*e.g*., hepatitis C, multiple sclerosis, Parkinson’s disease, spinal cord injury, polytrauma, etc.). Moreover, the vast majority of specialty care within the VA is pyramid referral-based, similar to epilepsy’s. Therefore, our findings will be relevant to VA care delivery more generally and may inform future decisions about the organization of VA care for other chronic conditions.

This project will provide information regarding possible targets for future interventions, including possible approaches based on the RC framework. Findings will provide VA clinicians with an assessment of the quality of VA epilepsy care and suggest areas for further improvement. Moreover, findings will provide VA clinicans and researchers with information about the types of strategies that were used in the ECOE initiative, how RC was associated with those strategies, and which approaches were associated with improvements and which were not. Thus, this study will provide a foundation for the design and implementation of interventions to further improve VA epilepsy care. Findings from this study will also provide information to assist VA policy makers in projecting numbers of patients with epilepsy and adapting staffing levels to accommodate treatment for those individuals.

We believe that findings from this study will also be relevant in non-VA settings. The ongoing study by the Institute of Medicine on the public health dimensions of the epilepsies (access, quality and education) supports concern regarding quality of and access to care broadly in the United States [39]. To date, there has been only one comprehensive assessment of the quality of epilepsy care in the US. That study, conducted by our team, was of a single healthcare system in one city, and suggested that there is much room for improvement [19]. The proposed study will provide the impetus to begin quality assessment for epilepsy care by examining quality in a national integrated healthcare system, putting the VA once again at the forefront of epilepsy care research.

Finally, our findings may have broad implications for healthcare delivery and system redesign. The methods of the proposed research are rooted in the evolving sciences of patient safety and process improvement and implementation science. In the era of national healthcare reform, these fields are increasingly important to improving patient outcomes and reducing unnecessary harm and utilization. Implications from this study may also inform the literature these fields [21,36].

### Strengths

Our study applies state-of-the-art quantitative and qualitative approaches to a topic of high interest inside and outside the VA. It is the first of its kind to measure the quality of epilepsy care in a national integrated healthcare system at three points in time, while concomitantly collecting data to describe the specific changes in structure and processes of epilepsy care in the VA that result from the ECOE initiative. The inclusion of the RC measure further strengthens the usefulness of this study by also contributing to implementation science and allowing us to learn what works. Our methods are rigorous. We not only triangulate data sources by using national databases and interview data from both clinicians and administrators, but also feedback our observations to individuals from whom the data is collected to maximize the validity of our analysis.

While cause and effect cannot be ascertained in this quasi-experimental study, we will have longitudinal measures that can be examined, in light of other changes in the field, to better rule out a cohort effect. As such, our study will provide evidence that can inform development of interventions and subsequent research to implement those interventions to improve quality and access to epilepsy care.

### Limitations

Although these data will provide important insights into VA care processes, the findings from this integrated healthcare system may not be generalizable to other settings. These findings can, however, be used to develop future research in other settings and further our understanding of relational coordination in healthcare systems of national scope.

## Conclusions

This innovative study will provide insight into the effectiveness of the ECOE hub-and-spoke model and explore the impact of restructuring epilepsy on VA care processes for Veterans with epilepsy. Moreover, the study will elucidate the role relational coordination plays in implementation of innovations designed to improve access to and quality of care.

### Ethical considerations

This study has been approved by VA IRB Central (study number IIR 11–067).

## Appendix

QUality Indicators in Epilepsy Treatment (QUIET)

Evaluation of Initial Seizure

1. All patients should have the results of at least one electroencephalography (EEG) reviewed or requested. If EEG was not performed previously, then an EEG should be performed.

2. All patients should have results of at least one magnetic resonance imaging (MRI) or computerized tomography (CT) scan reviewed or requested. Or, if a MRI or CT scan was not obtained previously, then a MRI or CT scan should be ordered (MRI preferred).

3. All patients should receive information on driving restrictions, safety, and injury prevention.

Initial Diagnosis & Treatment

4. If a patient is thought to have a diagnosis of epilepsy, then the diagnosis should include a best estimation of seizure types.

5. If the patient meets the criteria for epilepsy diagnosis (generally two unprovoked seizures), then seizure medication (SM) treatment should be discussed with and offered to the patient and caregivers.

6. If the patient is diagnosed with a seizure disorder/epilepsy and started on therapy, then monotherapy is preferred.

7. If the patient is a woman of childbearing potential (12-44 years old), then referral to a neurologist or an epilepsy specialist is indicated.

8. If a woman with epilepsy is of childbearing potential (12-44 years old), then she should receive information about the teratogenicity associated with treatment with valproate or topiramate and accept those risks prior to treatment.

9. If the patient is diagnosed with a seizure disorder/epilepsy, then during the visit the patient should receive information on:

•Driving restrictions, safety and injury prevention, diagnosis and treatment options including the importance of taking SMs as directed

•Triggers and other lifestyle factors that may affect seizure control (e.g. sleep deprivation, alcohol/drug use), and contraception and family planning

10. If a person with epilepsy is prescribed an SM that interacts with warfarin, then INR should be monitored within a week of any change in SM therapy, especially during polytherapy. Once the INR is stable, it should be monitored every four weeks.

Chronic Epilepsy Care

11. When a patient with epilepsy receives follow-up care, then an estimate of the number and types of seizures since the last visit and an assessment of drug side-effects should be documented.

12. When a patient with epilepsy receives follow-up care, then drug side-effects should be assessed and documented.

13. If the patient continues to have seizures after initiating treatment, then interventions should be performed. Options include:

•Compliance assessment/enhancement

•Monitor SM blood levels

•Increased SM dose

•Change SM dose

•Patient education regarding lifestyle modification

•Referral to higher level of epilepsy care

14. If a patient with epilepsy continues to have seizures after three months of care by a primary care provider, then further assessment by a neurologist should be conducted.

15. If a patient continues to have seizures after 12 months of appropriate care by a general neurologist, then the patient should receive a referral to an epilepsy specialist.

16. Patients with epilepsy should receive an annual review of information including topics such as:

•Chronic effects of epilepsy and its treatment including

•Drug side-effects, drug-drug interactions, and their effect on bone health,

•Contraception, family planning, and how pregnancy or menopause may affect seizures,

•Screening for mood disorders,

•Triggers and lifestyle issues that may affect seizures,

•Impact of epilepsy on other chronic and acute diseases,

•Safety issues (injury prevention, burns, driving restrictions, etc.)

•Other patient self-management issues

17. If the patient is on SM for 2 or more years, then providers should assess bone health.

18. Individuals receiving seizure medications should be screened for depression/suicide related behaviors (e.g., PHQ-9) initially, then 4-6 weeks (or the next clinic visit) after SM initiation and then at least yearly.

19. If a person with epilepsy is found to have evidence of a mood disorder (e.g., depression, anxiety), then s/he should receive treatment or a referral for mental health care.

Chronic Epilepsy Care for Women

20. If a woman with epilepsy is of childbearing potential (12-44 years old), then she should receive daily supplemental folate at a dose of at least 400 mcg.

21. If a woman with epilepsy is of childbearing potential (12-44 years old) and receives oral contraceptives in conjunction with an enzyme inducing SM, then decreased effectiveness of oral contraception should be addressed. (Higher doses of oral contraceptives, alternative birth control methods, or change SM may be needed).

22. Prenatal care for a woman with epilepsy should be co-managed by a neurologist and an obstetrician with experience in high risk pregnancy to assure that issues related to the impact of epilepsy and its treatment on the pregnancy are addressed.

Patient Generated Quality Indicator Statements

23. Providers should refer patients to local support groups or other resources to obtain psychosocial support.

24. Providers should encourage patients to become educated about epilepsy and to advocate for themselves in the health care system and with providers. For example, provide patients with written material about epilepsy, references to epilepsy foundation or epilepsy web sites.

25. Providers should communicate with patients about potential medication side effects, including cognitive, emotional, physical and sexual side effects.

26. Providers should give referrals to social services to assist with employment, negotiating through the Social Security Disability Insurance, insurance and alternative transportation for patients who cannot drive.

27. Providers should discuss the complexity of epilepsy treatment and explain that each patient responds to medications differently and that they may need to try several different medications before they find out what works best for that individual.

## Abbreviations

AED: Anti-epileptic Drugs; COE: Center of excellence; CPRS: Computerized patient record system; ECOE: Epilepsy Center of Excellence; OEF/OIF: Operation enduring freedom/operation iraqi freedom; OPC: Outpatient clinic records; PBM: Pharmacy benefits management; PRC: Polytrauma rehabilitation center; PTE: Post-traumatic epilepsy; PTF: Patient treatment file; SM: Seizure medication; TBI: Traumatic brain injury; VA: Veterans health administration; VistA Web: Veterans health information systems technology and architecture web.

## Competing interests

The authors have no competing interests to declare.

## Authors’ contributions

MJP obtained funding for this study. LKL, JAP, EPF, PHN, and HJL contributed to the design of the study. MJP supervised the manuscript. KKM, JAP, LKL, EPF, PHN, and HJL contributed to the manuscript. All authors contributed to the final manuscript. All authors read and approved the final manuscript.

## Supplementary Material

Additional file 1 Abstraction Tool. Click here for file
